# Sonodynamical reversion of immunosuppressive microenvironment in prostate cancer via engineered exosomes

**DOI:** 10.1080/10717544.2022.2044937

**Published:** 2022-03-03

**Authors:** Dingyi Wang, Zhuo Wan, Qian Yang, Jianmei Chen, Yunnan Liu, Fan Lu, Jie Tang

**Affiliations:** a Department of Ultrasound, The First Medical Center of Chinese PLA General Hospital, Beijing, China; b Department of Hematology, Tangdu Hospital, Fourth Military Medical University, Xi’ an, People’s Republic of China; c Department of Health Medicine, The Fourth Medical Center of Chinese PLA General Hospital, Beijing, China; d Department of Ultrasound, Tangdu Hospital, Fourth Military Medical University, Xi’an, People’s Republic of China; e Department of Biochemistry and Molecular Biology, State Key Laboratory of Cancer Biology, Fourth Military Medical University, Xi’an, People’s Republic of China

**Keywords:** Prostate cancer, exosomes, sonodynamic therapy, immunoadjuvant, synergistic effect

## Abstract

Prostate cancer (PCa) responds poorly to routine immunotherapy due to the tumor immunosuppressive microenvironment. Here, we describe an ultrasound-based drug delivery strategy to stimulate potent anti-tumor immunity via exosomes encapsulated with sonosensitizers Chlorin e6 (Ce6) and immune adjuvant R848, namely Exo^Ce6+R848^. Exo^Ce6+R848^ was constructed by simple co-incubation of Ce6 and R848 with HEK 293T cell-derived exosomes. The properties of exosomes were not affected after loading Ce6 and R848, and the exosomes were accumulated in the tumor site after intratumoral injection. *In vitro* and *in vivo* assays showed that ultrasonic irradiation enhanced R848-mediated DCs maturation when Exo^Ce6+R848^ was engulfed by DCs, as demonstrated by the upregulated expression of CD80 and CD86. Furthermore, these engineered exosomes together with ultrasound irradiation could synergistically reprogram macrophages from an immunosuppressive M2-like phenotype to an anti-tumor M1-like phenotype, further activating effector T cells and reverting the immunosuppressive microenvironment. The exosome delivery strategy not only supplies a paradigm for overcoming side effects of systemic delivery of Ce6 and R848, but also offers an effective combination regimen of cancer immunotherapy.

## Introduction

Prostate cancer (PCa) is termed as the second most frequent cancer and the fifth leading cause of cancer death among men in 2020 (Siegel et al., 2021). Androgen deprivation treatment (ADT) is the standard treatment for advanced PCa. However, a majority of patients will inevitably develop into castration-resistant prostate cancer (CRPC) after ADT (Bansal et al., 2021; Runcie & Dallos, 2021; Stultz & Fong, 2021). In comparison to melanoma and non-small cell lung cancer (NSCLC), trails in PCa with immune checkpoint blockade (ICB) have yielded disappointing results (Kwon et al., 2014; Antonarakis et al., 2020), which is at least partially attributed to tumor immunosuppressive microenvironment (Zhao et al., 2019; Stultz & Fong, 2021) and deficiency in maturation of dendritic cells (DCs) (Wculek et al., 2020; Jhunjhunwala et al., 2021). Thus, activating DCs maturation and reversing immunosuppression might boost PCa immunotherapy.

Sonodynamic therapy (SDT) employs ultrasound in combination with sonosensitizers (such as Chlorin e6, Ce6) to induce ROS locally (McHale et al., 2016). Besides, SDT was reported to improve antigen presentation ability, which may promote the immune activation and infiltration of T cells (Peng et al., 2018; Huang et al., 2021). The combination of SDT with an immunoadjuvant, such as the agonist of toll-like receptors 7 and 8 (TLR7/8), resiquimod (R848), is hypothesized to induce stronger anti-tumor immunity. However, both the sensitizing agent and R848 have disadvantages such as poor water solubility, easy aggregation, low bioavailability, poor tumor specificity, and fast clearance *in vivo*, etc. Thus, it is a critical issue to explore a promising local drug delivery strategy for codelivery both sonosensitizers and R848.

Exosomes have attracted considerable attention for local drug delivery (Li et al., 2021). Exosomes are extracellular vesicles secreted by cells, with the diameter ranging from 30 to 150 nm. As promising drug carriers for targeted delivery, the encapsulated chemotherapeutic drugs, RNA and natural products, are protected from degradation by enzymes or other extracellular conditions. Compared with common drug carriers, such as liposomes, micelles, and polymersomes, exosomes have the advantage of inherent stability, biocompatibility, biological barrier permeability, low toxicity, and low immunogenicity (You et al., 2018; Pullan et al., 2019; Nezhadi et al., 2021).

Here, we proposed an exosome-based strategy to locally deliver Ce6 and R848 at tumor sites. Our study revealed that locally delivered Ce6 and R848 activated DCs and reverted the tumor suppressive microenvironment in the xenograft model upon ultrasound irradiation.

## Materials and methods

### Cell lines

HEK293T cells, DC2.4 and RAW264.7 cells were originally purchased from ATCC (Manassas, VA). The mouse PCa cell line RM-1 cells provided by Procell Life Science & Technology Co., Ltd. (Wuhan, China), DC2.4 and RAW264.7 cells were carefully expanded and maintained in RPMI 1640 Medium (Hyclone, Logan, UT) supplemented with 10% fetal bovine serum (FBS) (Exocell Bio, Shanghai, China**)** and 1% penicillin–streptomycin solution (Hyclone, Logan, UT). HEK293T cells were cultured in DMEM medium (Hyclone, Logan, UT) with 10% FBS and 1% antibiotics. The cells were incubated under a saturating humidity atmosphere at 37 °C containing 5% CO_2_.

### Animal tumor model

The male C57BL/6 mice (8–12 weeks old, 21–24 g) from the experimental animal center of the Air Force Military Medical University (Xi’an, China) were housed and processed in accordance with the Institutional Animal Care and Use Committee in the Air Force Military Medical University. 1 × 10^6^ RM-1 cells suspended in 100 μL of PBS were subcutaneously implanted into the mice at the right hind leg to establish the animal tumor model.

### Exosome isolation

Exosomes from the donor HEK293T cells were isolated by ultracentrifugation in the study. For ultracentrifugation, cell culture supernatants were collected after 48 h of serum-free culture, followed by centrifugation at 300×*g* for 5 min to remove dead cells and then at 3000×*g* for 25 min to eliminate residual cellular debris. The resulting supernatants were then filtered through 0.22 µm filters, followed by additional centrifugation at 100,000×*g* for 3 h. The sediment was resuspended in PBS, and the mixture was additionally centrifuged at 100,000×*g* for 1 h to obtain relatively pure exosomes. The exosomes were resuspended in PBS and stored at −80 °C till use.

### Preparation and characterization of Exo^R848+Ce^6

Exo^R848+Ce^6 was synthesized using co-incubation technique. Chlorin e6 was purchased from Cayman Chemical (Ann Arbor, MI). Resiquimod (R848) was purchased from MedChemExpress (Monmouth Junction, NJ). Briefly, 30 µL of R848 (2.5 µg/µL) and 30 µL of Ce6 (5 µg/µL) were gently incubated with 300 µL purified exosomes (10^9^ particles/mL) for 2 h at 37 °C. Free R848 and Ce6 were removed by another round of exosome isolation.

To verify the characterization of Exo^Ctrl^ and Exo^R848+Ce6^, the exosomes were added onto the grid and stained with 2% uranyl acetate, followed by imaging with the transmission electron microscope (JEM2000EX TEM, JEOL Ltd., Tokyo, Japan). Isolated exosomes were diluted to 500 ng/mL and subjected for size distribution analysis by Nanoplus.

### Loading capacity of exosomes

The concentrations and loading capacity of exosomes were quantified using characteristic UV–vis absorptions by the Nanodrop-2000 spectrophotometer (Thermo Scientific, Waltham, MA). Briefly, Ce6 and R848 dry products were dissolved in DMSO with concentration of 10 mM and stored at −20 °C. To determine the drug concentration at maximum loading rate, different doses of Ce6 and R848 (30 µL) were added to 300 µL purified exosomes (10^9^ particles/mL) for incubating 2 h at 37 °C and then centrifuged at 100,000×*g* for 60 min to remove the excess free drug. The supernatant containing the free drug was collected and absorption intensity at the maximum absorption peak (*A*_2_) was measured. The same dose of drug was added to DMSO and absorption intensity (*A*_1_) was measured. The loading rate was calculated by the following equation: loading rate=(*A*_1_ – *A*_2_)/*A*_1_×100%.

### Western blotting

Total protein from HEK293T cells, exosomes, and DC2.4 was homogenized in RIPA Lysis Buffer (Beyotime Biotechnology, Shanghai, China), and the protein concentrations were measured using a BCA Protein Assay Kit (Thermo, Waltham, MA). Equal amounts of protein samples (15 μg) were electrophoresed in 5% and 12% SDS-PAGE (120 V for stacking gel and 160 V for separation gel) and then transferred onto polyvinylidene fluoride (PVDF) membranes. Subsequent to blocking by 5% nonfat milk for 1 h, the membranes were incubated with primary antibodies, anti-GM130 (11308-1-AP, Proteintech, Rosemont, IL), anti-TSG101 (ab83, Abcam, Cambridge, UK), anti-CD9 (ab92726, Abcam, Cambridge, UK), anti-GAPDH (D110016-0100, BBI Life Sciences, Shanghai, China), or anti-Hsp70 (ab2787, Abcam, Cambridge, UK) overnight at 4 °C. After washing three times in TBST, the membranes were cultured with anti-rabbit (7074, CST, Boston, MA) and anti-mouse (7076, CST, Boston, MA) secondary antibodies corresponding to the primary antibodies at room temperature for 1 h.

### Exosomes tracking *in vivo*

Exosomes were labeled with DiI/DiR (at the final concentration of 10 µM, Invitrogen, Carlsbad, CA) by incubating with the dye at the ratio of 500:1 in volume for 30 min. Unlabeled free dyes were then removed by centrifugation after washed with PBS. Mice were then injected with 200 µL labeled exosomes via tail vein or intratumor. For fluorescence imaging *in vivo*, different tissues from the mice that injected with DiR-labeled exosomes were harvested. IVIS^®^ Lumina II *in vivo* imaging system was used for exosome localization in the whole body and individual organs. For test of the sliced section, DiI-labeled exosomes were prepared similarly before tail vein or intratumor injection. Four hours after injection, mice were sacrificed and the organs and tumors were fixed with 4% paraformaldehyde for 15 min and again washed with PBS twice. The cell nuclei were stained with Hoechst 33342 (1:1000, Beyotime Biotechnology, Shanghai, China) for 10 min. The whole process was kept from light. The fluorescence signal for the labeled exosomes and the blue nuclei were visualized by Nikon A1 Spectral Confocal Microscope (Nikon, Tokyo, Japan).

To further verify the distribution of exosomes *in vivo*, exosomes were electroporated with one OD miR54 at 700 V/150 mF in 4 mm wide electroporation cuvettes. Relative expression of miR54 in different issues was analyzed by qPCR as described below.

### Optimal parameters for SDT

To investigate the efficacy of SDT based on different concentrations of Exo^Ce6^ and intensities of ultrasound, the cell viability of RM-1, DC2.4, and RAW264.7 cells was determined with a Cell Counting Kit-8 (CCK-8) assay (SK2060, Coolaber, Beijing, China). The cells cultured in 96-well plates were incubated with different concentrations of Exo^Ce6^ (5 × 10^8^, 10^9^ or 2 × 10^9^ particles/mL) for four hours, then exposed to ultrasound of various intensities (0.1, 0.5, or 1.0 W/cm^2^) at a frequency of 1 MHz, using a 20% duty cycle, for 5 min. After treatment, 100 µL of cell suspension was incubated with 10 µL CCK-8 solution for 2 h at 37 °C in a 5% CO_2_ atmosphere. The absorbance of the samples at a wavelength of 450 nm was measured using a microplate reader (MULTISKAN MK3, Thermo Fisher Scientific, Waltham, MA). The parameters were used for the following *in vitro* experiments.

### ROS detection

ROS generation in DC2.4 was measured using the fluorescent probe dihydroethidium (DHE, Beyotime Biotechnology, Shanghai, China). DC2.4 with density of 10^4^ cells per mL was seeded on glass bottom cell culture dish and subjected to PBS, Exo^Ctrl^, Exo^R848^, Exo^Ce6^+US, and Exo^R848+Ce6^+US. The exosome concentration was 10^9^ particles/mL, the groups treated with Ce6 were exposed to ultrasound at a frequency of 1 MHz, a power density of 0.1 W cm^−2^, using a 20% duty cycle, for 5 min as verified above. After this, cells were harvested, washed with serum-free RPMI medium and incubated with 5 µM DHE at 37 °C for 30 min. DHE intensity field was measured by Nikon A1 Spectral Confocal Microscope and FACS Canto II (BD Biosciences, Franklin Lakes, NJ), with an excitation wavelength of 300 nm and emission wavelength of 610 nm.

### *In vivo* tumor treatment

Tumor development over time was estimated using the equation: tumor volume = length × width × height × π/6. When the tumors had reached an average size of 30 mm^3^, animals were randomly distributed into five groups (the PBS group, the Exo^Ctrl^ group, the Exo^R848^ group, the Exo^Ce6^+US group, and the Exo^R848+Ce6^+US group, *n* = 5 in each group). On the 10th, 12^th^, and 14th day of subcutaneous tumor bearing in mice, 2 × 10^9^ particles/kg body weight of exosomes (10^9^ particles/mL), loaded with 0.2 mL Ce6 (5 µg/µL, loading rate 70.08%) or/and 0.2 mL R848 (2.5 µg/µL, loading rate 58.56%) were injected into tumors in the different groups. Following induction of anesthesia (intraperitoneal injection of 1% pentobarbital sodium), the groups treated with Ce6 were exposed to ultrasound 2 h later since injection at a frequency of 1 MHz, a power density of 2.0 W cm^−2^, using a 20% duty cycle, for 5 min. Notably, exosomes became obviously accumulated at the tumor site since the beginning of intratumor injection. For therapeutic reason, ultrasound was conducted and repeated three times. The therapeutic effectiveness in each studied group was evaluated by measuring the size of the primary tumors over time. Besides, some of the tumor tissues were collected from different groups of mice on the second day after the last administration for further analysis. At the end of the experiment, the tumors were dissected and weighed from the sacrificed mice.

### Real-time qPCR for gene expression analysis

Total RNA of the cells or tumor tissues was extracted with Trizol reagent (Invitrogen, Carlsbad, CA) according to the manufacturer’s instructions. Reverse-transcription was conducted using PrimeScript First-Strand cDNA Synthesis Kit (Takara, Beijing, China) for mRNA and miRcute First-Strand cDNA Synthesis Kit (Tiangen Biotech, Beijing, China) for miRNA. Gene expression was analyzed using PrimeScript RT Master Mix (Roche, Basel, Switzerland) or miRcute miRNA qPCR Kit (Roche, Basel, Switzerland). GAPDH and U6 were served as an internal control in mice for standardization between samples and relative RNA levels of target genes. The fold change of target gene was calculated using the 2^–ΔΔCt^ method. The specific primers for individual genes are shown in the Supplementary Table 1.

### H&E staining and immunofluorescence

Mice were sacrificed and tumor tissues were dissected. Tissues were fixed in 4% paraformaldehyde, dehydrated with sucrose solutions, embedded with paraffin, and then cut into slides. The sections were then stained with hematoxylin and eosin.

After incubation with 5% bovine serum albumin (BSA) for 1 h, the sections were incubated with primary antibody obtained from Servicebio (Woburn, MA): anti-CD11c rabbit (GB11059), anti-CD3 rabbit (GB130140), anti-CD4 rabbit (GB130642), anti-CD8 rabbit (GB13014), anti-Foxp3 Rat (GB13445), anti-F4/80 rabbit (GB11027), anti-CD206 rabbit (GB13438), and anti-CD86 rabbit (GB13585) overnight at 4 °C in a wet, dark box. Subsequently, the sections were incubated with the secondary antibody (HRP-conjugated goat anti-rabbit IgG, GB23303, Servicebio (Woburn, MA); CY5 conjugated goat anti-rabbit IgG, GB27303, Servicebio (Woburn, MA); HRP conjugated goat anti-rat IgG, GB23302, Servicebio (Woburn, MA)) for 1 h at room temperature. Cell nuclei were stained with DAPI (G1012, Servicebio, Woburn, MA). The sections were washed with PBS and then observed with a Nikon A1 Spectral Confocal Microscope (Nikon, Tokyo, Japan).

### Flow cytometry analysis

To study the antitumor immune effect, tumors were cut into small pieces and digested in 5 mL collagenase type I at 37 °C for 50 min. The single cell suspensions were washed with Red Cell Lysis Solution. For surface marker analysis, the cells were stained with APC-conjugated-anti-mouse CD11c (N418, Proteintech, Rosemont, IL), FITC-conjugated-anti-mouse CD3 (145-2C11, Proteintech, Rosemont, IL), PerCP-conjugated-anti-mouse CD4 (GK1.5, Biolegend, San Diego, CA), APC-conjugated-anti-mouse CD8 (53-6.7, Proteintech, Rosemont, IL), FITC-conjugated-anti-mouse F4/80 (ab60343, Abcam, Cambridge, UK), APC-conjugated-anti-mouse CD206 (C068C2, Biolegend, San Diego, CA), and PerCP-conjugated-anti-mouse CD86 (GL-1, Biolegend, San Diego, CA) at 37 °C for 30 min. For intracellular staining, the cells were permeabilized with Fix&Perm Kit (Multi Sciences Biotech, Hangzhou, China) for 30 min according to the manufacturer’s instructions followed by re-staining with APC-conjugated-anti-mouse Foxp3 (3G3, Proteintech, Rosemont, IL), and finally analyzed by flow cytometry. All samples were performed by the FACS Canto II (BD Biosciences, Franklin Lakes, NJ) or CytoFLEX (Beckman Coulter, Brea, CA) cytometers. Data were further analyzed by FlowJo V10 software.

### Statistics

All values were presented as mean ± SEM. One-way ANOVA was used to compare the differences among three or more groups while multiple comparisons were performed by Tukey’s post hoc test (GraphPad Prism 8.0; GraphPad Software, La Jolla, CA). *p* Values of <.05 indicated statistical significance.

## Results

### Construction and characterization of Exo^Ce6+R848^

**Figure 1.**
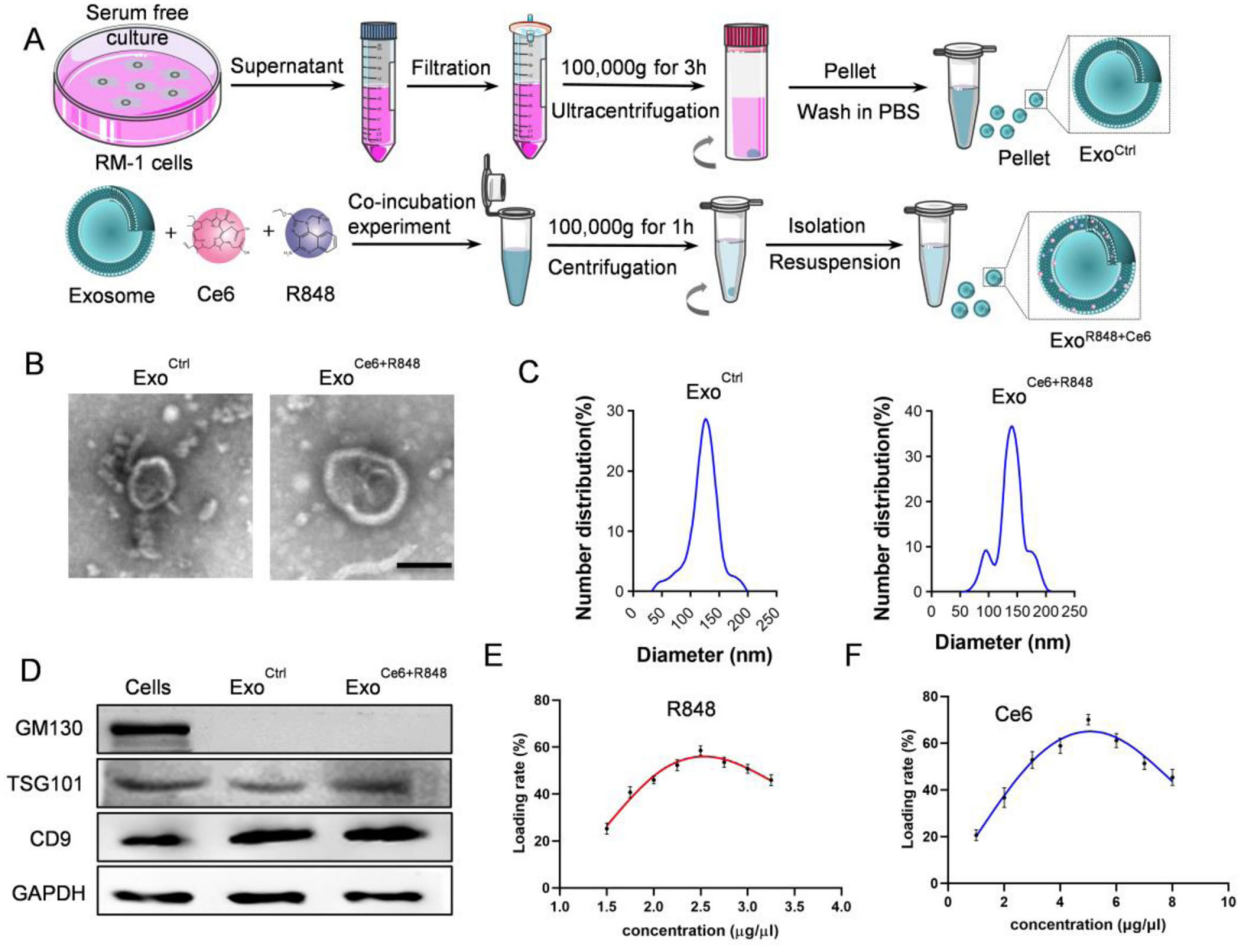
Construction and characterization of Exo^Ce6+R848^. (A) Schematic representation of exosomes isolation and Exo^Ce6+R848^ construction. (B) Representative TEM (transmission electron microscope) image of the Exo^Ctrl^ and Exo^Ce6+R848^ (scale bar = 100 nm). (C) Size distribution of the Exo^Ctrl^ and Exo^Ce6+R848^. (D) Western blot analysis of exosomal markers in cells, Exo^Ctrl^ and Exo^Ce6+R848^. (E) Drug loading efficiency of R848 that was prepared with various concentrations. (F) Drug loading efficiency of Ce6 that was prepared with various concentrations.

In this study, we used HEK 293 T cell-derived exosomes and constructed Exo^Ce6+R848^ by co-incubation technique (Figure 1(A)), which displayed the typical exosome morphology as revealed by transmission electron microscopy (Figure 1(B)). Nanoparticle tracking analysis showed that the size distribution of Exo^Ctrl^ and Exo^Ce6+R848^ ranged from 30 to 150 nm and the size of Exo^R848+Ce6^ was slightly larger than the parental Exo. As shown in Figure 1(C), there were two peaks for the engineered exosomes. However, they remain in the range of exosomes. Moreover, western blot analysis revealed the exosomal inclusive marker TSG101 and CD9 were found to be expressed in both Exo^Ctrl^ and Exo^Ce6+R848^, while the exclusive marker GM130 was absent (Figure 1(D)). These data demonstrated that exosomes were successfully extracted and their properties were not affected after loading Ce6 and R848.

Ce6 and R848 concentrations were determined by the absorption measurement (Figure S1A). The UV–vis spectrum revealed that Ce6 and R848 had the maximum absorption peak at 400 nm and 250 nm (Figure S1B,D), respectively. There was a good linear relationship between the absorbance and concentration of Ce6 and R848 (Figure S1C,E). When the concentration of R848 was 2.5 μg/μL and the concentration of Ce6 was 5 μg/μL, loading efficiency was 58.56% and 70.08% in 10^9^/mL exosome solution, respectively (Figure 1(E,F)).

### Optimization of exosome injection routes

**Figure 2.**
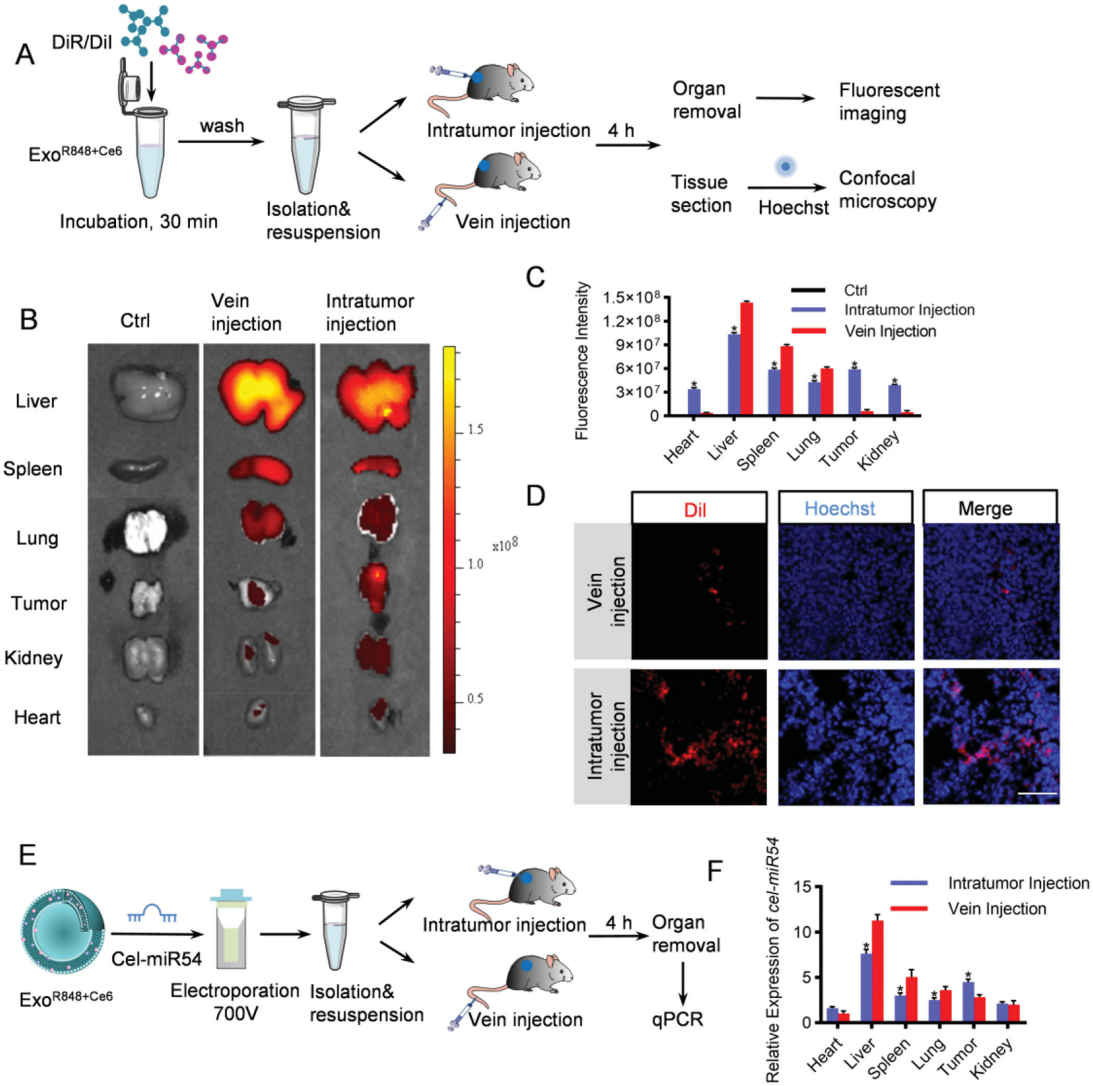
*In vivo* distribution of exosomes. (A) Schematic diagram of the experimental procedure. Mice were injected with DiR/DiI-labeled exosomes to monitor the distribution of exosomes. (B) Representative *ex vivo* fluorescent images of various organs and tumor issues in mice by vein injection or intratumor injection with DiR-labeled exosomes. *n* = 5 mice. (C) Quantification of (B). (D) Confocal microscopic images of the localization of DiI-labeled exosomes in tumor issues. Scale bar = 100 μm, *n* = 5 mice. (E) Schematic diagram of the experimental procedure. Mice were injected with cel-miR54-loaded exosomes to monitor the distribution of exosomes. (F) qPCR analysis of expression of cel-miR-54 in various organs and tumor issues in mice. U6 served as an internal control. The expression of cel-miR-54 in various organs was normalized to the heart by vein injection. Data are expressed as mean ± SEM of six independent biological samples. *p<.05, intratumor injection versus vein injection.

To track the efficient distribution of exosomes to major organs (heart, liver, spleen, lung, and kidney) and the tumor issue, we injected DiR/DiI-labeled exosomes into the tumor directly or via tail vein (Figure 2(A)). As expected, both *ex vivo* fluorescent imaging and confocal microscope analysis showed that the liver, spleen, and lung were the dominant targets of exosomes with the strongest fluorescence intensity both when injecting into the tumor directly and via tail vein. However, compared with tail vein injection, the distribution of exosomes in tumor issues increased significantly via intratumoral injection (Figure 2(B–D), Figure S2). To further confirm the results, mice were injected with cel-miR54-loaded exosomes as cel-miR54 has no homolog in mice (Figure 2(E)). qPCR analysis of cel-miR54 abundance showed the enrichment of exosomes in tumor tissues can be significantly increased by intratumoral injection compared with tail vein injection (Figure 2(F)).

### Exo^R848+Ce6^-mediated enhancement of antigen presentation and macrophages repolarization *in vitro*

To determine the optimal SDT parameters of *in vitro* experiment to make it have certain killing activity on tumor cells, while not affecting the viability of immune cells, we incubated RM-1, DC2.4, and RAW264.7 cells with three different concentrations of Exo^Ce6^ and ultrasonic intensities. The results showed that no obvious cytotoxicity of ultrasound treatment was observed for all the cell types. Furthermore, stimulation with 10^9^ particles/mL Exo^Ce6^ and the intensity of 0.1 W/cm^2^ resulted in the highest relatively viability in DC2.4 (90.22%) and RAW264.7 cells (84.96%) compared with tumor cells (70.59%) (Figure S3). Confocal images *in vitro* revealed efficient endocytosis of exosomes both by DCs and RM-1 cells (Figure S4).

**Figure 3.**
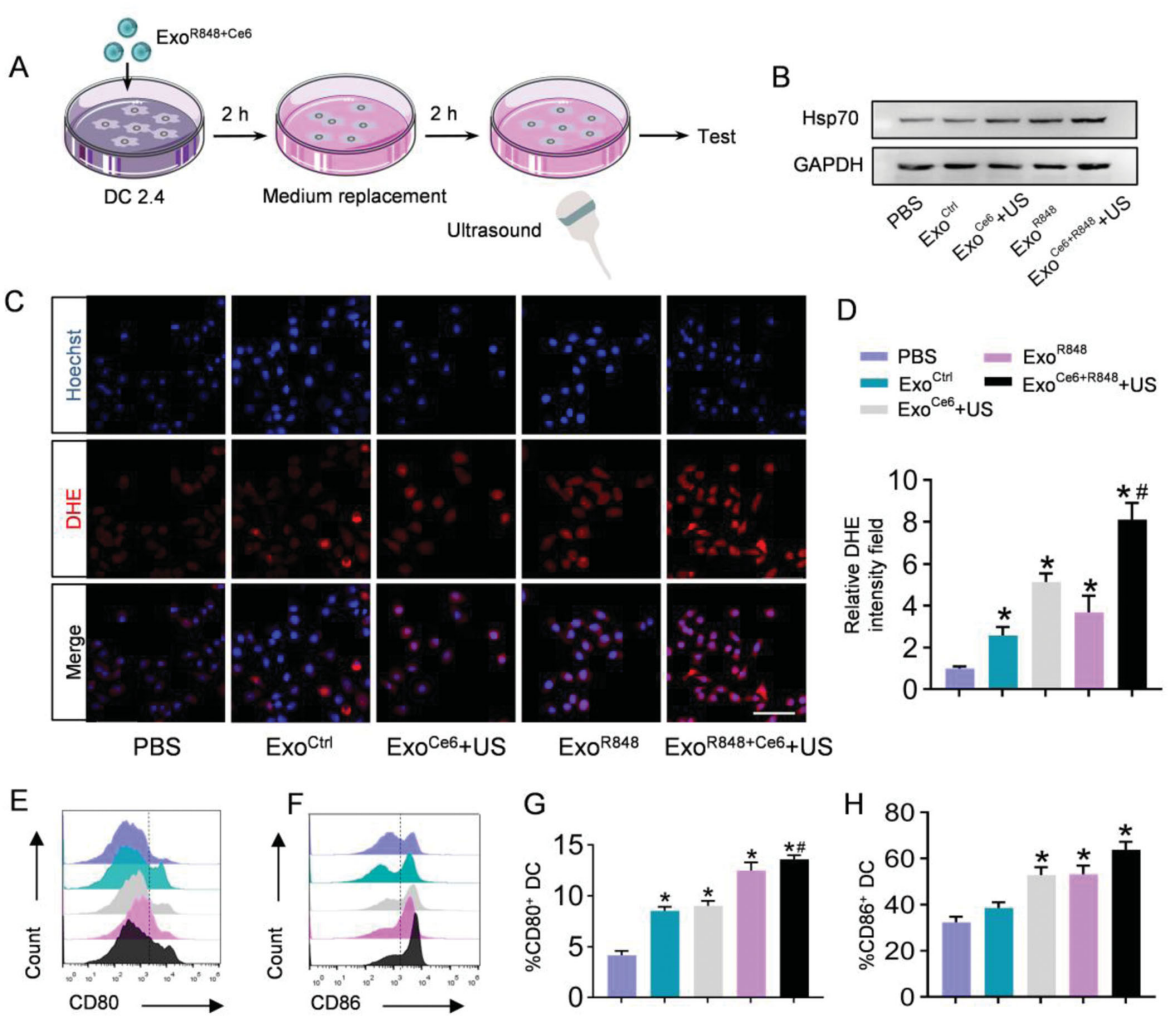
Exo^R848+Ce6^-mediated enhancement of antigen presentation of DC2.4 *in vitro*. (A) Schematic illustration of the experimental procedure. (B) Western blot analysis of Hsp70 in DC2.4 cells after treated with different formulations. GAPDH served as internal control. (C) Total ROS generation in DC2.4 cells after different treatments was detected by confocal microscope, scale bar = 100 μm. (D) Quantification of (C). (E, F) Flow cytometric analysis assessing the expression of CD80 and CD86 on DC2.4 cells in the various treatment groups. (G, H) Quantification of the enhanced maturation of DCs. Data are expressed as mean ± SEM of five independent biological samples. *p<.05, Exo^Ce6^+US, Exo^R848^, Exo^Ce6+R848^+US versus Exo^Ctrl^; ^#^*p*<.05, Exo^Ce6+R848^+US versus Exo^R848^.

The mechanism of Exo^R848+Ce6^+US enhancement of DCs maturation was first determined by detecting intracellular ROS levels using DHE. As shown in Figure 3(C), after Exo^R848^ or Exo^Ce6^+US treatment, abundance of ROS was generated in these groups, as verified by the strong red fluorescence in cells. The ROS intensity of Exo^R848+Ce6^+US group was the strongest of all (Figure 3(D)). ROS generation in cells was also quantified by flow cytometric analysis, further suggesting the significantly highest ROS generation of Exo^R848+Ce6^+US group in all the groups (Figure S5). We also evaluated the expression of Hsp70 on the surface of DCs after different treatment groups to further enhance its antigen presentation ability. As shown in Figure 3(B), Exo^R848^ and Exo^Ce6^+US could increase Hsp70 expression. Moreover, the combination treatment exhibited an obvious effect in comparison with other groups. Based on the above results, the synergistic effect which induced the DCs maturation to express the co-stimulatory molecules CD80/CD86 was verified by flow cytometry. As shown in Figure 3(E–H), the Exo^R848+Ce6^+US group resulted in a much higher level of CD80 and CD86 expression than other treatment groups.

To study the effect of Exo^R848+Ce6^+US on the macrophage polarization, interleukin-4 was first added to induce M2 macrophage polarization, followed by incubation with various treatments. Compared with the PBS and Exo^Ctrl^ group, Exo^R848^, Exo^Ce6^+US, and Exo^R848+Ce6^+US could decrease the expression of M2-related genes (*Mrc-1*, *Fizz-1*, and *Il-10*) and increase the expression of M1-related genes (*iNos*, *Il-6*, and *Il-1β*), indicating a polarization from the M2 phenotype to M1 phenotype (Figure S6).

### Enhanced antitumor efficacy and inflammatory response of Exo^Ce6+R848^+US on PCa tumor-bearing mice

**Figure 4.**
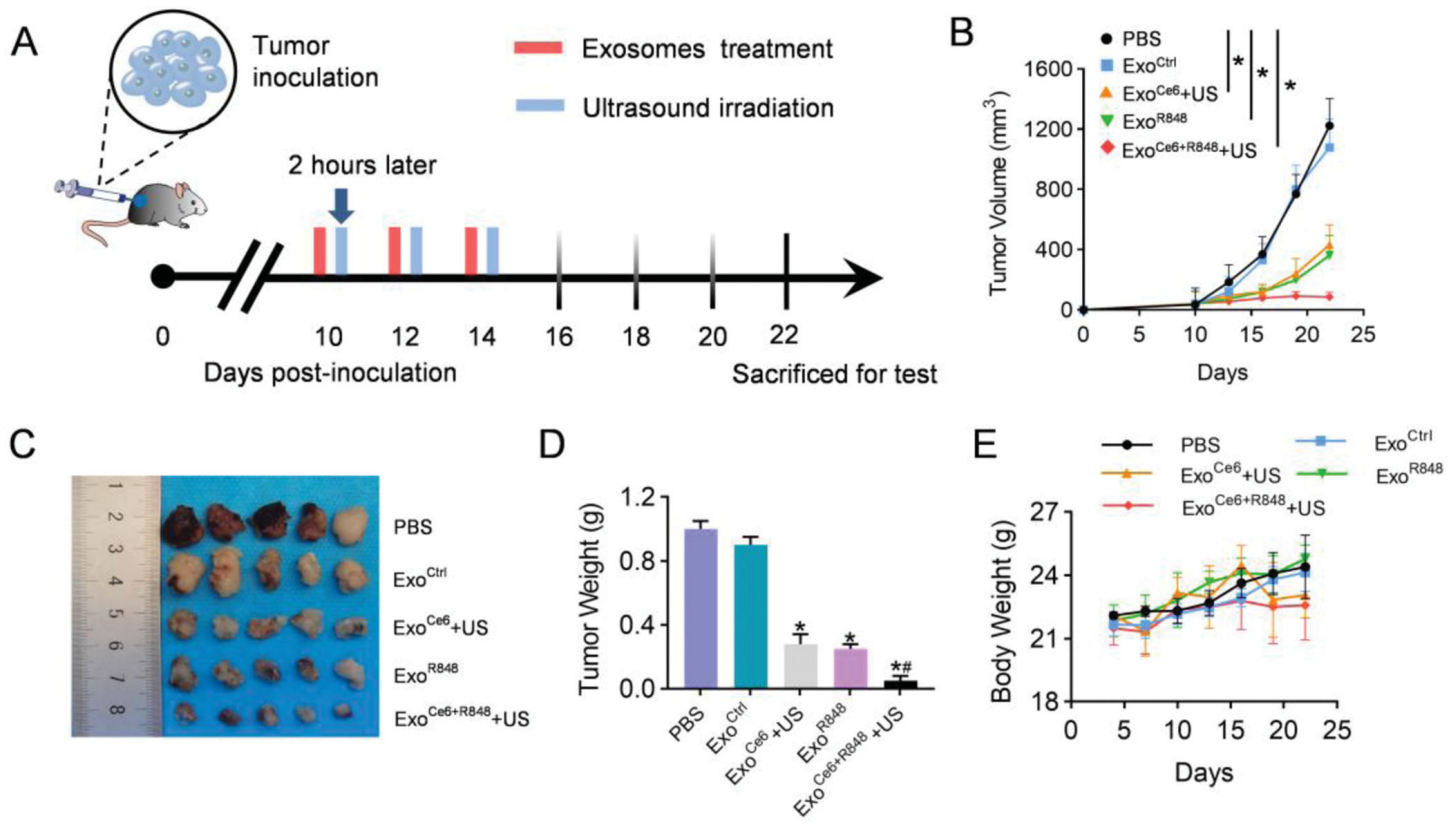
Therapeutic effects of Exo^Ce6+R848^+US on the growth of transplanted PCa tumor. (A) Schematic PCa tumor model and experimental design. 2 × 10^9^ particles/kg body weight of exosomes were intratumorally injected three times at an interval of two days. Insonation (1 MHz, 2.0 W cm^−2^, 20% duty cycle, 5 min) was applied two hours after administration. Tumor volume and body weight were recorded every three days until day 12 after the first treatment. (B) Mean tumor growth curves of primary tumor that underwent various treatments. Tumors were excised and examined on day 12 after the first treatment for (C) and (D). (E) Fluctuation curve of mice body weight during the postoperative treatment time period. *n* = 5 mice. Data represent mean ± SEM (*n* = 5). *p<.05, Exo^Ce6^+US, Exo^R848^, Exo^Ce6+R848^+US versus Exo^Ctrl^; #*p*<.05, Exo^Ce6+R848^+US versus Exo^R848^.

Animal studies were performed in mice bearing PCa to evaluate the synergistic anticancer effect of R848 and SDT *in vivo* (Figure 4(A)). As shown in Figure 4(B–D), all exosomes loading Ce6 or/and R848 showed obvious therapeutic effects, and tumor growth was most inhibited in mice treated with Exo^Ce6+R848^+US. On the 8th day after the last treatment, tumor volume in the Exo^Ce6+R848^+US was 86 ± 32 mm^3^. In comparison, tumors grew to 1224 ± 179 mm^3^, 1079 ± 188 mm^3^, 434 ± 132 mm^3^, and 362 ± 132 mm^3^ in the PBS group, Exo^Ctrl^ group, Exo^Ce6^+US group, and Exo^R848^ group, respectively (Figure 4(B,C)). Regardless of different treatments, the body weights of mice in all groups remained stable (Figure 4(E)).

The synergistic immunologic adjuvant and SDT based on exosomes were further explored on tumor tissue sections. As shown in Figure S7, tumor tissues from animals treated with PBS and Exo^Ctrl^ had fewer blood vessels and less inflammatory infiltration. In contrast, the blood vessels and inflammatory cells in tumor tissues from animals receiving Exo^Ce6^+US, Exo^R848^, or Exo^Ce6+R848^+US increased obviously. Moreover, tumor tissue from animal treated with Exo^Ce6+R848^+US showed the most significant increase in blood vessels and inflammatory cells.

**Figure 5.**
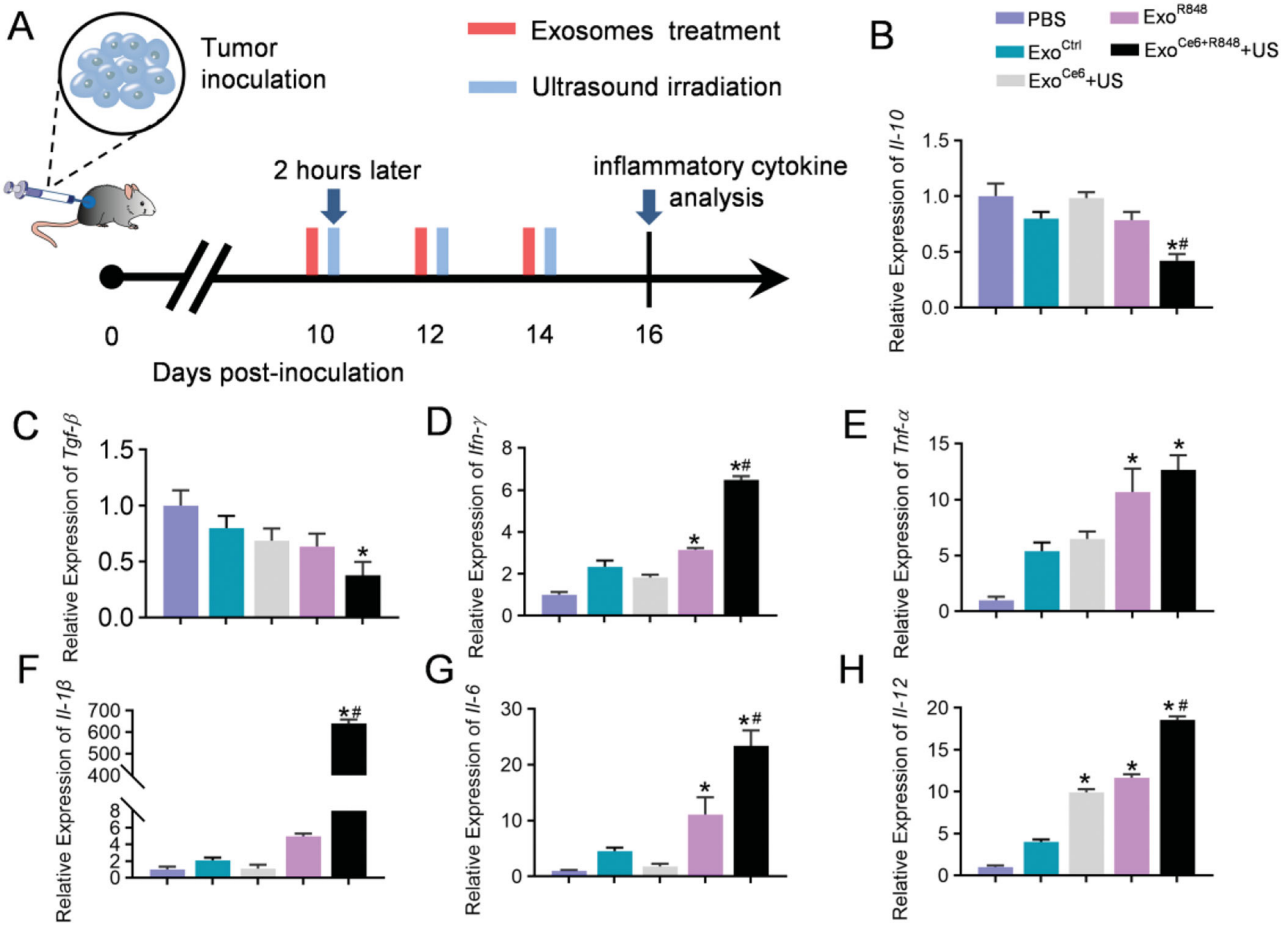
Effects of engineered exosomes on inflammatory cytokine. (A) Schematic diagram of the experimental procedure. 2 × 10^9^ particles/kg body weight of exosomes were intratumorally injected three times at an interval of two days. Insonation (1 MHz, 2.0 W cm^−2^, 20% duty cycle, 5 min) was applied two hours after administration. Tumors were excised for inflammatory cytokine analysis on day 16 after inoculation RM-1 cells. (B–H) Relative expression level of *Il-10*, *Tgf-β*, *Ifn-γ*, *Tnf-α*, *Il-1*, *Il-6*, and *Il-12* in tumor tissues with different treatments (*n* = 5). Data are expressed as mean ± SEM, one-way ANOVA, *p<.05, Exo^Ce6^+US, Exo^R848^, Exo^Ce6+R848^+US versus Exo^Ctrl^; ^#^*p*<.05, Exo^Ce6+R848^+US versus Exo^R848^.

To further investigate the immune cytokine storm induced by systemic immunity, inflammatory cytokines in tumor issues from mice receiving different treatments were analyzed by qPCR (Figure 5(A)). The Exo^Ce6+R848^+US therapy could obviously downregulate the secretion of the anti-inflammatory cytokine *Il-10* and *Tgf-β* in the tumor microenvironment (Figure 5(B,C)). At the same time, the combination therapy exhibited a pleasing pro-inflammatory effect, leading to 6.48-, 12.67-, 640.98-, 23.45-, and 18.56-fold upregulation of *Ifn-γ*, *Tnf-α*, *Il-1β*, *Il-6*, and *Il-12* in comparison with the control group (Figure 5(D–H)).

**Figure 6.**
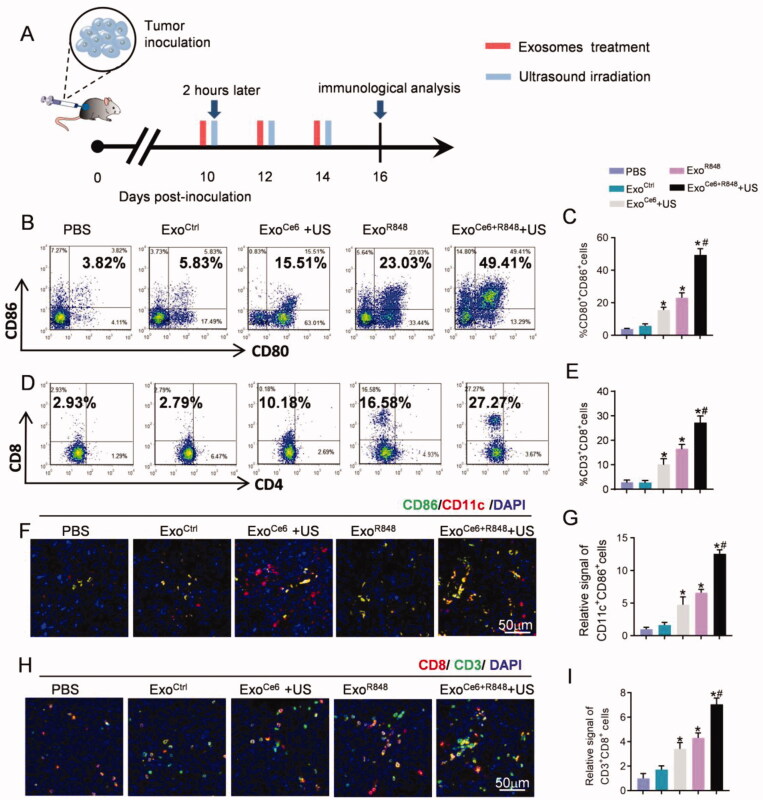
Exosomes in each treatment group for activating DCs and anti-tumor T-cell immune response by flow cytometric analysis and immunofluorescence. (A) Schematic diagram of the experimental procedure. Exosomes (10^9^ particles/mL) were injected three times at an interval of two days, and their dose was 2 × 10^9^ particles/kg body weight per injection. Insonation (1 MHz, 2.0 W cm^−2^, 20% duty cycle, 5 min) was applied two hours after administration. Tumors were excised for immunological analysis on day 16 after inoculation RM-1 cells. Flow cytometric analysis of (B) proportion of mature DCs (CD80^+^CD86^+^) over CD11c^+^ DCs and (D) CD8^+^ T cells over CD3^+^ T cells at the tumor tissues with different treatments. (C, E) Statistical results of (B) and (D), respectively (*n* = 5). (F) Immunofluorescence staining of CD11c^+^ CD86^+^ in tumor sections after different treatments. (H) Immunofluorescence staining of CD3^+^ CD8^+^ in tumor sections after different treatments. (G, I) Statistical results of (F) and (H), respectively (*n* = 5). Data are expressed as mean ± SEM, one-way ANOVA, *p<.05, Exo^Ce6^+US, Exo^R848^, Exo^Ce6+R848^+US versus Exo^Ctrl^; ^#^*p*<.05, Exo^Ce6+R848^+US versus Exo^R848^.

Accordingly, Exo^Ce6+R848^+US treatment resulted in a highest number of mature DCs (49.41%±3.35%) among all groups, which are 23.03%±3.01% in the Exo^R848^ group, 15.51%±1.65% in the Exo^Ce6^+US group, 5.83%±1.20% in the Exo^Ctrl^ group, and 3.84%±0.47% in the PBS group, respectively (Figure 6(B,C,F,G)). To further investigate whether the Exo^Ce6+R848^+US therapy induced effective anti-tumor T cell responses, the numbers of tumor-infiltrating T cells in various treatment groups were measured. In comparison with the rare tumor infiltration of CD3^+^CD8^+^ T cells in the PBS group (2.93%±0.81%) and Exo^Ctrl^ group (2.79%±0.97%), the effector T cells were obviously increased via treatments of other exosomes. In particular, Exo^Ce6+R848^+US treatment showed the best effect to promote the tumor infiltration of CD3^+^CD8^+^ T cells (27.27%±2.74%) (Figure 6(D,E,H,I)). In contrast, the number of CD3^+^CD4^+^ T cells did not change significantly after different treatment groups (Figure 6(D), Figure S8).

**Figure 7.**
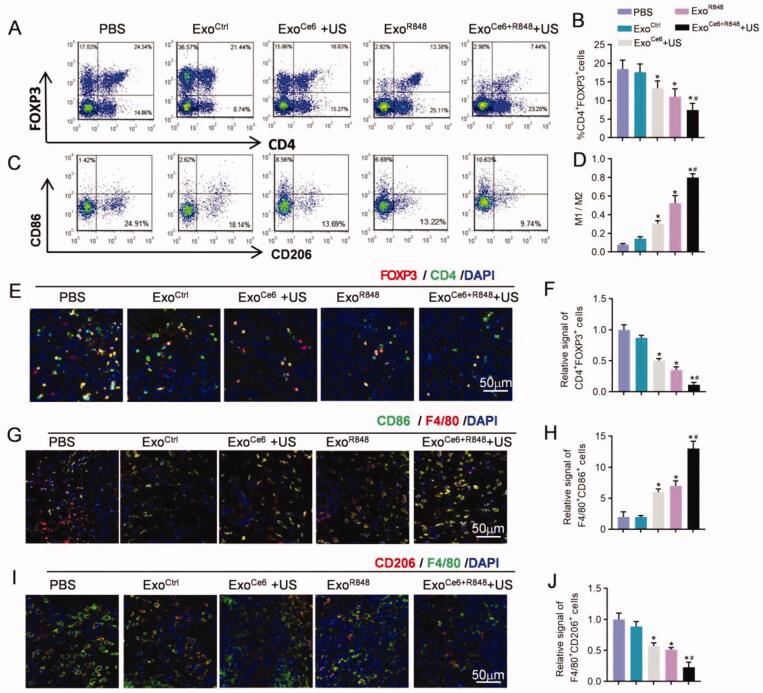
Enhanced immunity and macrophage polarization of Exo^Ce6+R848^ for reeducating tumor immunosuppressive microenvironment. (A) Flow cytometric analysis of proportion of CD4^+^ Foxp3^+^ T cells (representing Treg cells) and M1 (CD86^+^) and M2 (CD206^+^) macrophages over F4/80^+^ cells (C) at the tumor tissues after different treatments. (B, D) Statistical results of (A) and (C), respectively (*n* = 5). (E) Immunofluorescence staining of Foxp3^+^ CD4^+^ in tumor sections after different treatments. (G) Immunofluorescence staining of F4/80^+^ CD86^+^ in tumor sections after different treatments. (I) Immunofluorescence staining of F4/80^+^ CD206^+^ in tumor sections after different treatments. (F, H, and J) Statistical results of (E), (J) and (I), respectively (*n* = 5). Data are expressed as mean ± SEM, one-way ANOVA, *p<.05, Exo^Ce6^+US, Exo^R848^, Exo^Ce6+R848^+US versus Exo^Ctrl^; ^#^*p*<.05, Exo^Ce6+R848^+US versus Exo^R848^.

In contrast, Exo^Ce6+R848^ and ultrasonic radiation synergistically reduced the populations of Tregs (CD4^+^Foxp3^+^) and increased M1/M2 ratio (F4/80^+^CD86^+^/F4/80^+^CD206^+^) as determined by flow cytometry and immunohistochemistry (Figure 7). Compared with the PBS group, the number of Tregs was 0.87, 0.50, 0.35, and 0.11-fold lower in the Exo^Ctrl^, Exo^Ce6^+US, Exo^R848^, and Exo^Ce6+R848^+US group, respectively (Figure 7(A,B,E,F)). The combination therapy showed a greater decline than the Exo^R848^ group. Besides, as shown in Figure 7(C,D,G–J), the M1/M2 ratio of Exo^Ce6^+US and/or Exo^R848^ groups exhibited a significant increase compared with the PBS and Exo^Ctrl^ group. Notably, the ratio of Exo^Ce6+R848^+US group was 0.81 ± 0.04 while the value of the Exo^R848^ group was 0.53 ± 0.12, which indicated an enhanced M1 polarization effect of the combination therapy.

## Discussion

In this study, we demonstrated that engineered Exo^Ce6+R848^ can inhibit the progression of PCa upon ultrasound irradiation in tumor-bearing mouse model. Mechanistically, Exo^Ce6+R848^ could stimulate robust maturation of tumor-infiltrating DCs, promote M1 macrophages polarization and pro-inflammatory cytokines production, and decrease anti-inflammatory factors, further inhibiting Tregs and activating effector T cells in TME. As professional APCs, DCs play a central role in initiating and regulating tumor immune responses. Only mature DCs can migrate to tumor-draining lymph nodes to present antigenic peptides to CD8^+^ T cells and subsequently recognize and destroy cancer cells. Instead of allowing maturation of DCs and a DC-mediated antitumor response, DCs in tumors can be redirected toward a dysfunctional, tolerogenic, or even immunosuppressive phenotype (Pfirschke et al., 2017; Wculek et al., 2020; Jhunjhunwala et al., 2021). Therefore, conversion of tumor-infiltrating DCs from tolerogenic to immunogenic by the effective strategies is of great importance.

In this study, we efficiently incorporated Ce6 and R848 into the exosome membrane by co-incubation technique. Since both Ce6 and R848 are lipophilic, they can adhere to lipid bilayer of exosomes via co-incubation technique. Due to the lipophilic characteristics, R848 and Ce6 themselves could be rarely loaded inside the exosomes (which is similar as the cytosol) even if they penetrate into the exosomes. This passive drug loading method, which is relatively simple and straightforward, can maintain the membrane integrity of exosomes. HEK293T cells-derived exosomes were chosen for the reason that they were easy for manipulation, free of immunity, and widely used in animal models. Compared with liposomes and other polymeric nanoparticles, which have intricate shortcomings such as activation of immune system, low circulating capability, stability, and high toxicity (Elsharkasy et al., 2020; Herrmann et al., 2021), exosome-based delivery strategy can overcome these shortcomings with minimal toxicity, excellent biocompatibility, and an immune privileged status, which allows for decreased drug clearance according to their characteristics and natural origin (Batrakova & Kim, 2015; Pullan et al., 2019; Patil et al., 2020). In this study, exosomes were administrated via intratumor injection. The injection route has characteristics that ensure a long retention time at tumor site to increase the chance for extravasation due to the high permeability and retention effect (EPR). Besides, compared with vein injection, intratumor injection enhances the local concentration by reducing exosomes phagocytized by the mononuclear phagocyte system (MPS, e.g. liver and spleen).

Previous studies found that R848 increased the expression of immune genes including TLR7, IL6, Myd88, and IRF3, related to the TLR7/8 signaling pathway. These signaling pathways lead to activation of the transcriptional factor NF-κB, which, upon translocating to the nucleus, promoted the transcription of genes characteristic of DCs maturation, including genes for cytokines (e.g. IL-12, IL-1β, and TNF-α), co-stimulatory molecules (CD80, CD86), and MHC molecules (Zhou & Sun, 2015; Alam et al., 2018). However, prolonged exposure to R848 causes significant cytotoxicity and limits its continued use (Schmid et al., 2017; Michaelis et al., 2019). The proposed exosome delivery systems can limit dissemination of the drug into the systemic circulation following intratumoral injection.

Exosomes containing Ce6 engulfed by DCs can promote translocation of Hsp70 on to the cell surface and produce ROS under ultrasound. Previous studies have demonstrated that membrane-bound Hsp70 could promote the release of IL-6 and IL-1β as well as DCs maturation by the evaluation of CD80 and CD86 expression (Kuppner et al., 2001; Zhu et al., 2016; Zininga et al., 2018). Notably, excessive ROS production may result in premature death, while moderate ROS production is essential for DC activation and function (Grassi et al., 2007; Paardekooper et al., 2019). A recent study has demonstrated that ROS-mediated DNA oxidation enhanced immune recognition by DCs and DC-derived ROS was responsible for STING-initiated antitumor immune responses (Hu et al., 2021). Nonetheless, future researches need to explore the optimal intensity and frequency of ultrasound and dose of exosomes to induce DC maturation without killing immune cells at the tumor site.

Besides the direct impact on DCs, remodeling of the tumor microenvironment by the engineered exosomes could also be responsible for the observed therapeutic effects. In the tumor microenvironment, TAMs and Tregs are the most abundant immunosuppressive cells that inhibit T cells activation and proliferation. TAMs exhibit either an immunosuppressive M2-like phenotype, increasing production of anti-inflammatory factors IL-10 and TGF-β, or an anti-tumor M1-like phenotype, releasing pro-inflammatory cytokines IL-12, IL-1, IL-6, and TNF-α (Poh & Ernst, 2018; Chen et al., 2019; Pathria et al., 2019). The proposed strategy here repolarized TAMs from M2 to M1 phenotype to enhance cancer immunotherapy. The synergistic effect of TAM polarization and DCs maturation could unleash the suppressed T cells caused by the tumor microenvironment, activating effector T cells and improving their infiltration into tumors. Besides, it has been reported that tumor cells secreted the immunosuppressive molecules, such as IL-10 and TGF-β, inhibited the maturation of DCs and increased the production of Tregs (Tormoen et al., 2018; Hu et al., 2020). We found that tumor tissues of Exo^R848+Ce6^+US group displayed lower levels of these inhibitive factors, relieving the local tumor-mediated immunosuppression and generating an effective systemic immunity.

## Conclusions

In summary, we here confirmed that SDT and immunoadjuvant delivered via exosome-based delivery strategy not only evoked the DCs but also re-modulated the immunosuppressive tumor microenvironment in PCa tumor-bearing mouse model. The exosome delivery strategy not only supplies a paradigm for overcoming the systemic side effects of Ce6 and R848, but also offers a rational design of an effective combination regimen, which is promising for clinical translation.

## Supplementary Material

Supplemental Material

## Data Availability

The datasets used and/or analyzed during the current study are available from the corresponding author on reasonable request. Additional information available: additional Figures S1–S8 and Table S1.
