# Supplementary material for: Sonodynamical reversion of immunosuppressive microenvironment in prostate cancer via engineered exosomes
Source: Drug Deliv. 2022 Mar 3;29(1):702–13. doi: 10.1080/10717544.2022.2044937 (PMC8903759; doi:10.1080/10717544.2022.2044937)
Supplement: Supplemental Material [file IDRD_A_2044937_SM2713.doc]

**Supplemental Data**

**Sonodynamical reversion of immunosuppressive microenvironment in prostate cancer via engineered exosomes**

Dingyi Wang^1#^, Zhuo Wan^2#^, Qian Yang^1#^, Jianmei Chen^3^, Yunnan Liu^4^, Fan Lu^5^*, Jie Tang^1^*

1. Department of Ultrasound, the First Medical Center of Chinese PLA General Hospital, Beijing 100853, China.
2. Department of Hematology, Tangdu Hospital, Fourth Military Medical University, Xi’ an, 710038, People’s Republic of China.
3. Department of Health Medicine, the Fourth Medical Center of Chinese PLA General Hospital, Beijing 100048, China.
4. Department of Ultrasound, Tangdu Hospital, Fourth Military Medical University, Xi’an, 710038, People’s Republic of China.
5. State Key Laboratory of Cancer Biology, Department of Biochemistry and Molecular Biology, Fourth Military Medical University, Xi’an, 710032, People’s Republic of China.

# These authors contributed equally to this article.

* Correspondence author

**E-mail addresses:**

[lufan@fmmu.edu.cn](mailto:lufan@fmmu.edu.cn) (Fan Lu)

[txiner@vip.sina.com](mailto:txiner@vip.sina.com) (Jie Tang)

**Supplemental figures**


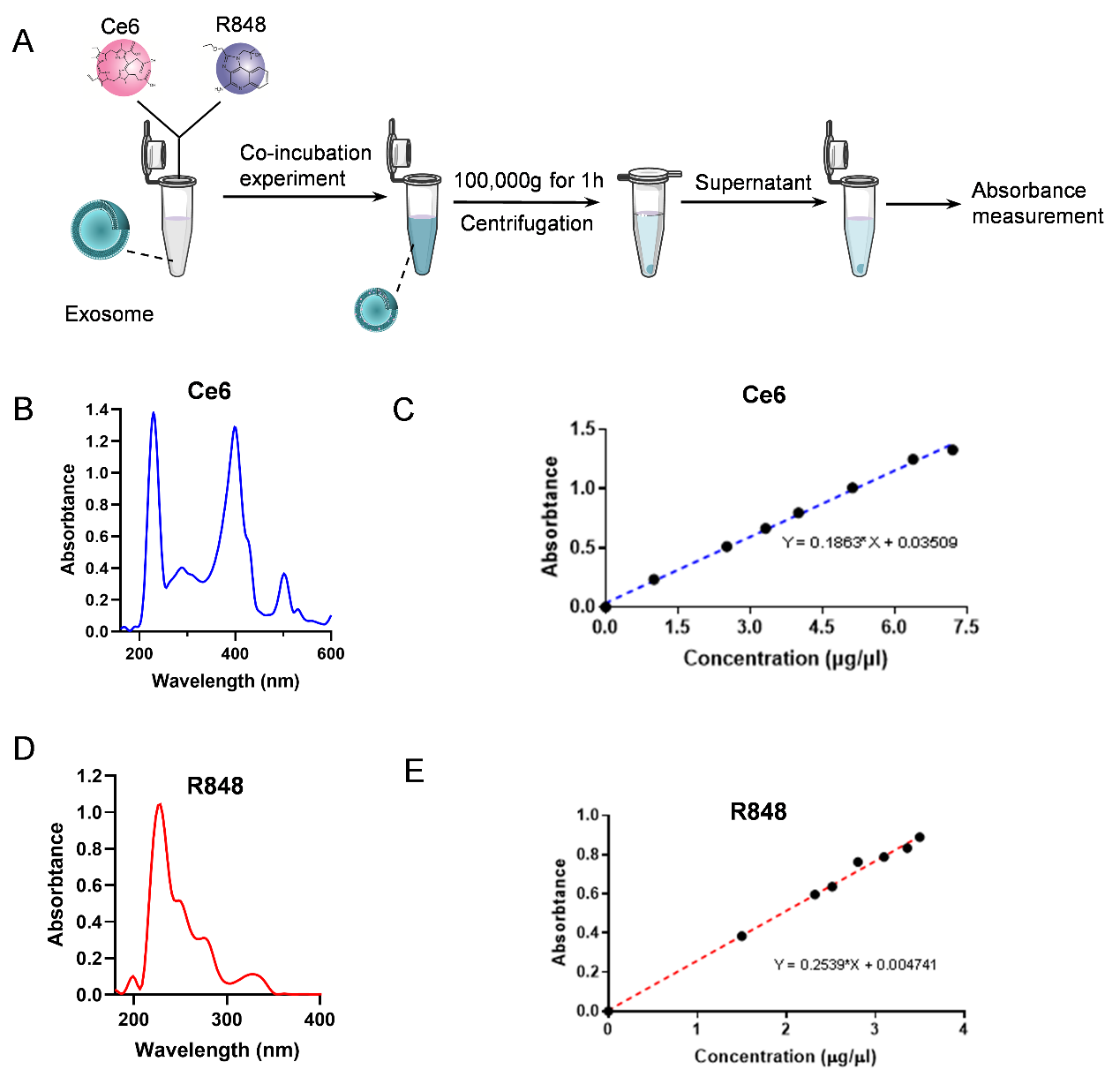


**Figure S1. Optical properties of Ce6 and R848.** (A) Schematic illustration of absorbance measurement. (B) The ultraviolet–visible absorption spectra of Ce6. (C) The relationship between the absorbance and concentration of Ce6. (D) The ultraviolet–visible absorption spectra of R848. (E) The relationship between the absorbance and concentration of R848.


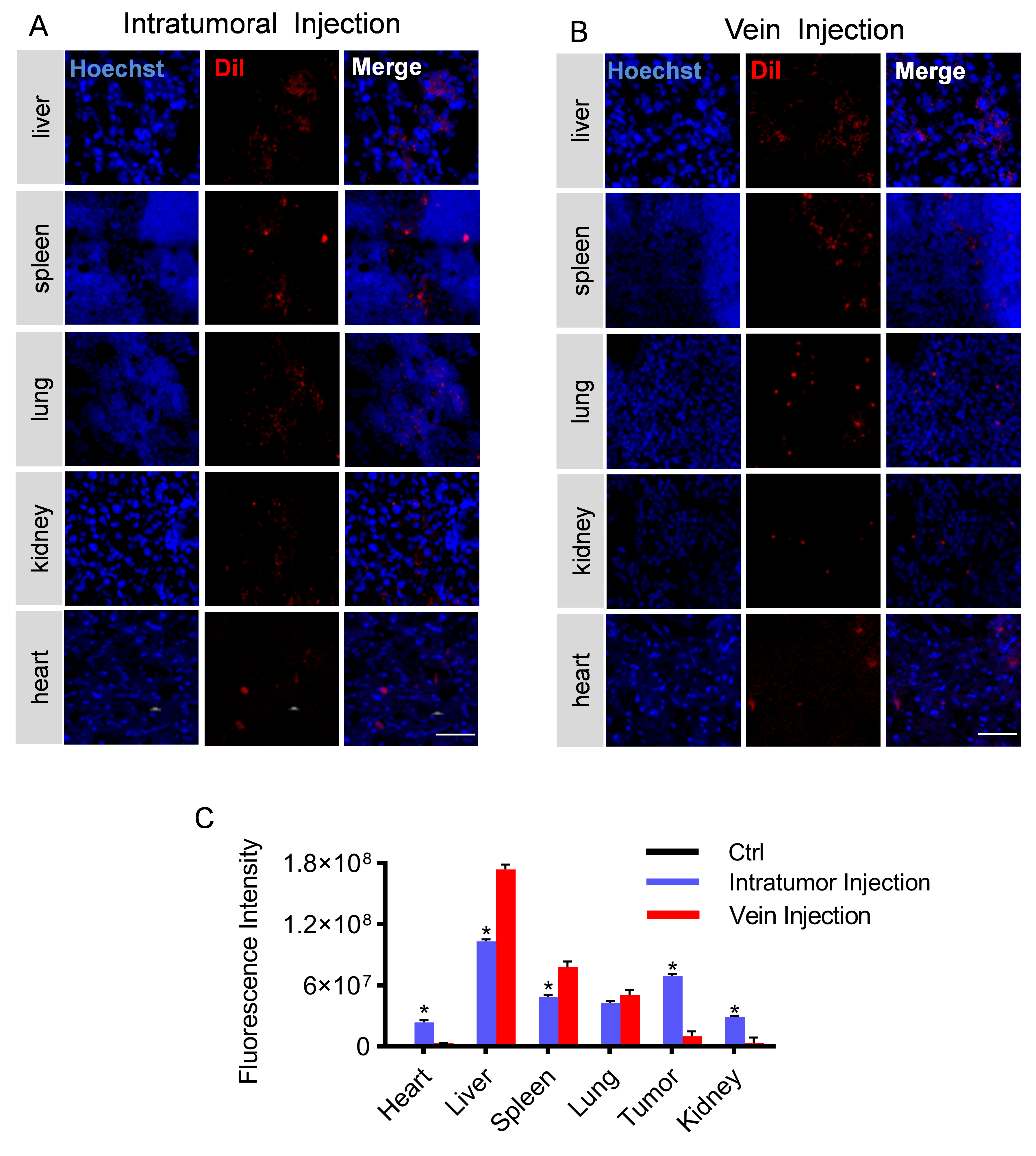


**Figure S2. In vivo distribution of exosomes.** (A, B) Confocal microscopic images of the localization of DiI-labeled exosomes by intratumor injection or vein injection in various organs (corresponding to Figure 2D). Scale bar = 100 μm, n=5 mice. (C) In vivo NIR intensity 2 h after different injection methods. **p*＜0.05, intratumor injection versus vein injection.

**
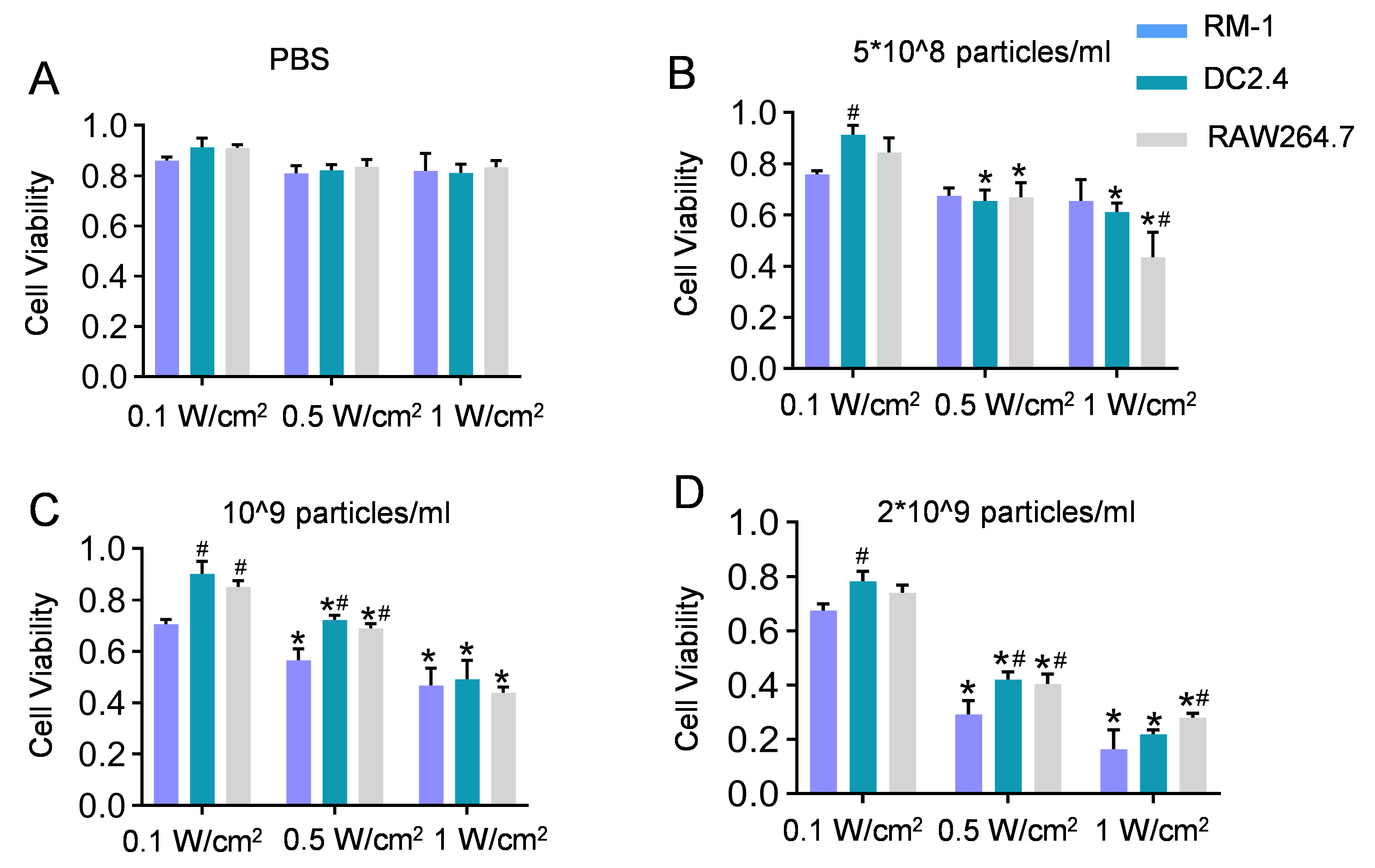
**

**Figure S3. Effects of SDT on cell viability.** Cell viability in the PBS, different concentrations of Exo^Ce6^ and intensities of ultrasound detected by CCK8 assay. Data are expressed as mean ± SEM, n=3. **p*＜0.05, 0.5 W/cm^2^, 1 W/cm^2^ versus 0.1 W/cm^2^; *^#^p*＜0.05, DC 2.4, RAW 264.7 versus RM-1.


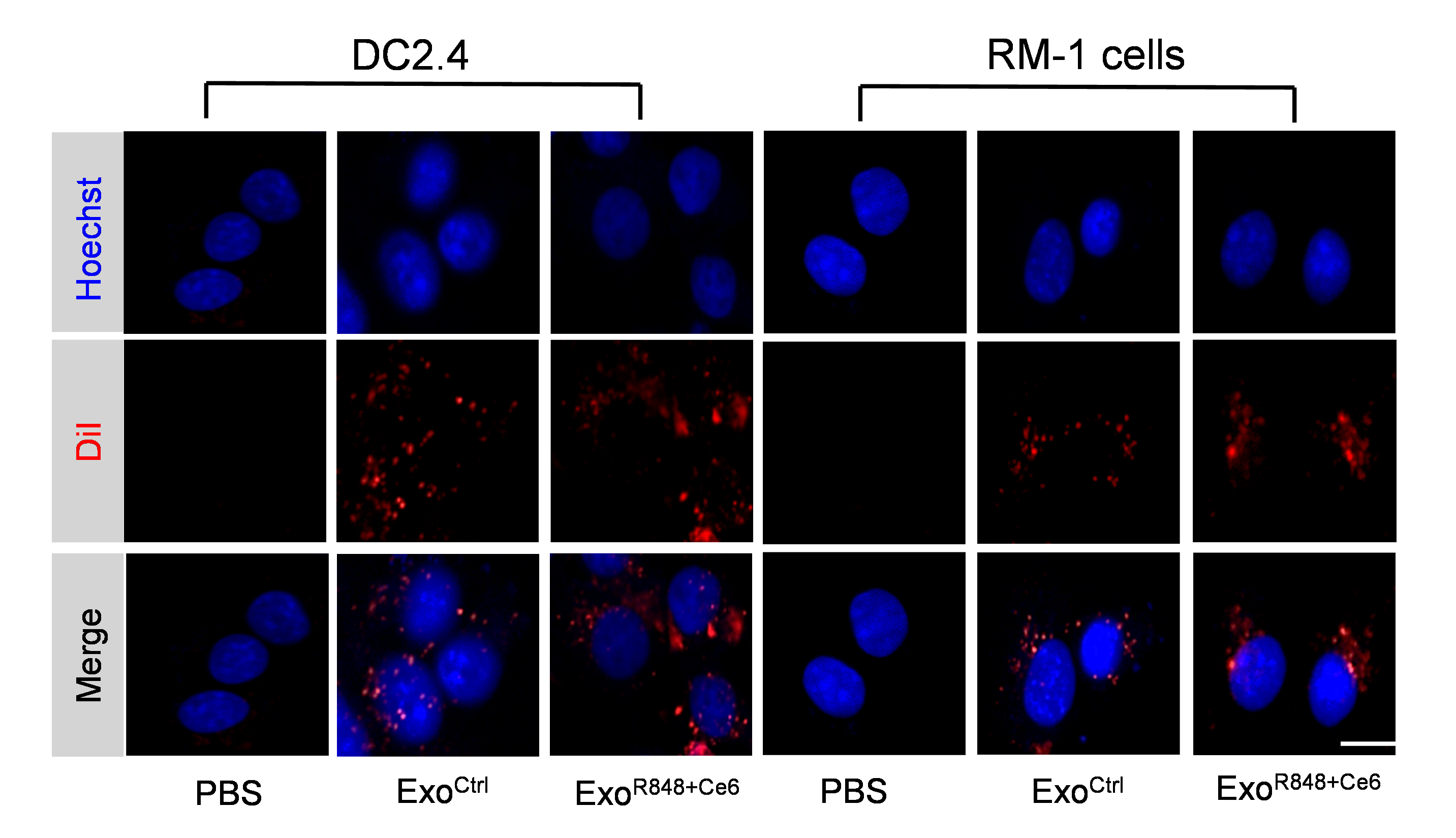


**Figure S4. Confocal microscopy images showing the endocytosis of exosomes in the DC2.4 and RM-1 cells.** The intracellular distribution of DiI-labeled exosomes was analyzed by confocal microscopy. Nuclei were counterstained with Hoechst. PBS was served as the negative control. Scale bar=5 μm.


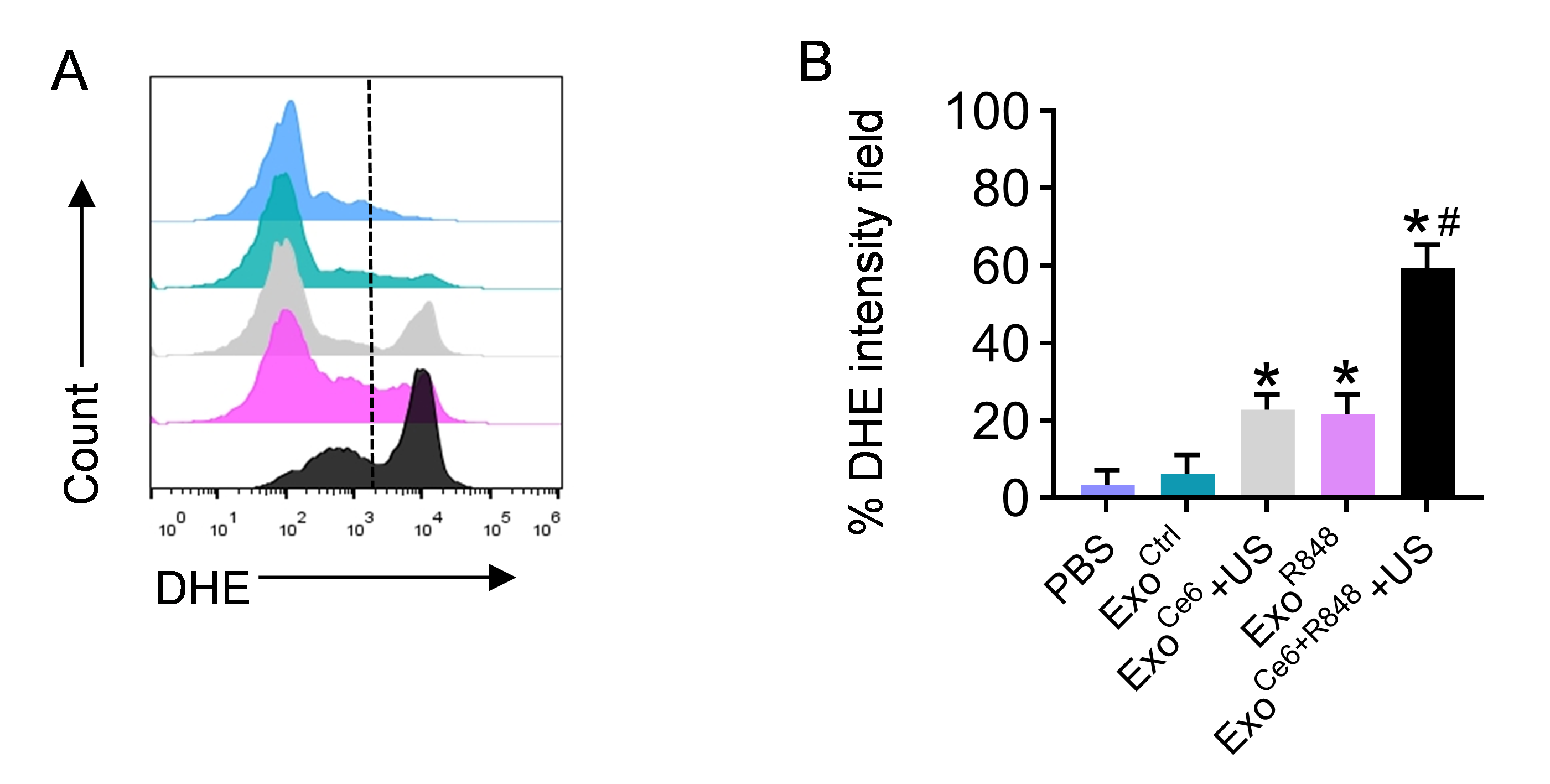


**Figure S5. ROS generation in vitro by flow cytometry.** Total ROS generation in DC2.4 cells after different treatments was detected by flow cytometry. Representative data of three different experiments. Data are expressed as mean ± SEM. **p*＜0.05, Exo^Ce6^+US, Exo^R848^, Exo^Ce6+R848^+US versus Exo^Ctrl^; *^#^p*＜0.05, Exo^Ce6+R848^+US versus Exo^R848^.


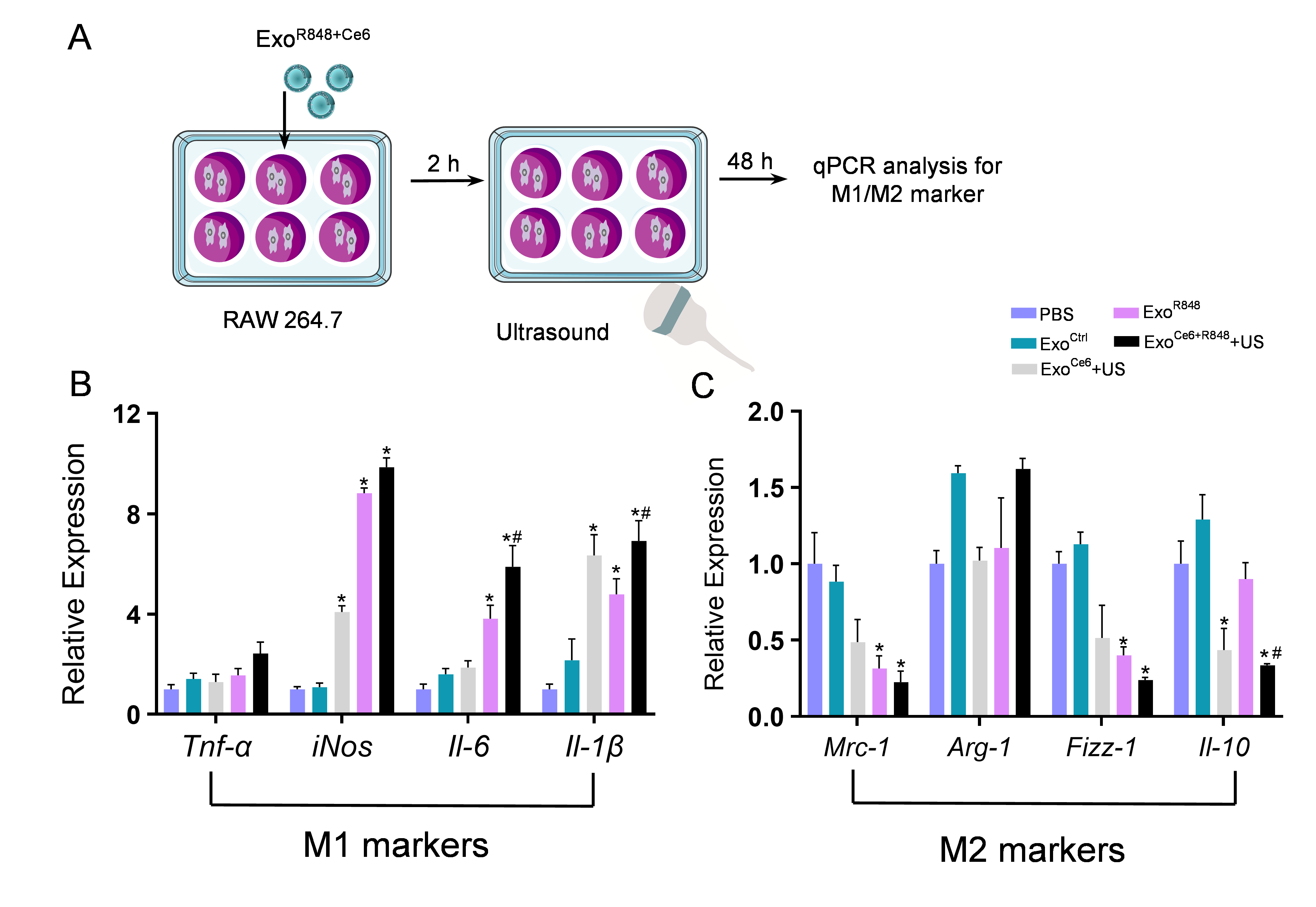


**Figure S6. In vitro M1 polarization efficiency of Exo^Ce6+R848^.** Expression levels of M1 or M2 markers in the RAW264.7 cells treated with different exosomes. Data are expressed as mean ± SEM of five independent biological samples. **p*＜0.05, Exo^Ce6^+US, Exo^R848^, Exo^Ce6+R848^+US versus Exo^Ctrl^; *^#^p*＜0.05, Exo^Ce6+R848^+US versus Exo^R848^.


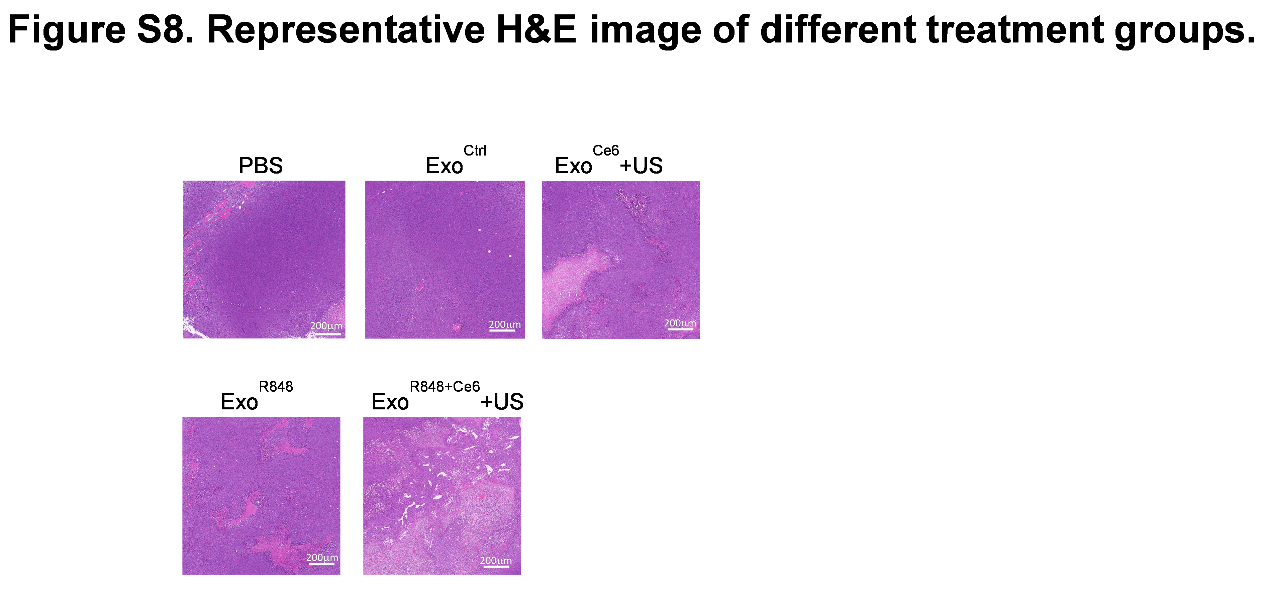


**Figure S7.** Representative H&E staining images of tumor-bearing mice tumor issues treated with PBS, Exo^Ctrl^, Exo^Ce6^+US, Exo^R848^ and Exo^Ce6+R848^+US, respectively.


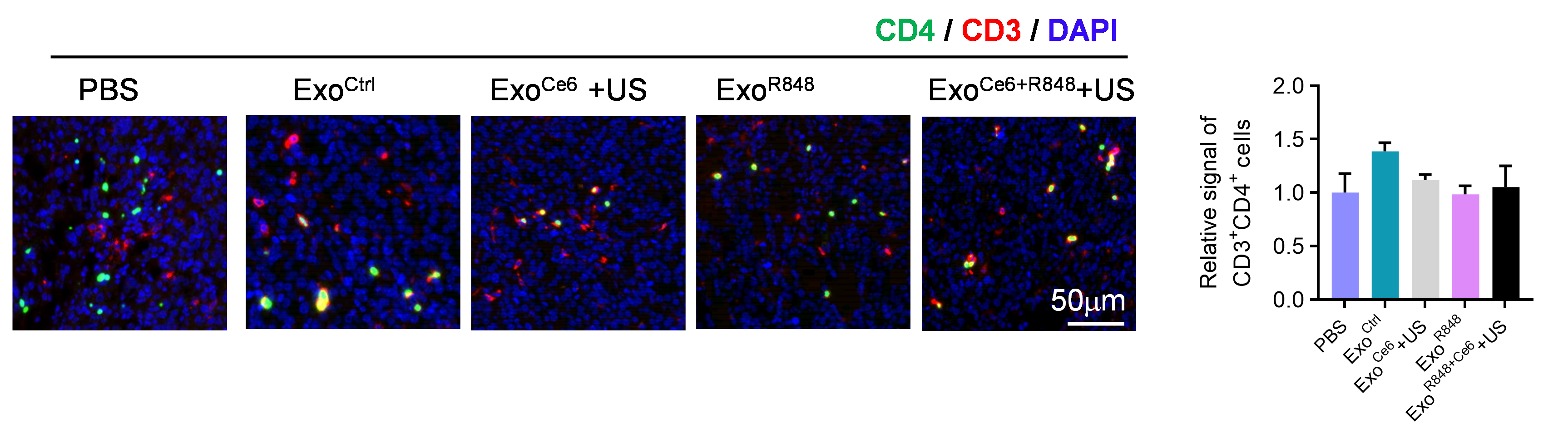


**Figure S8. In vivo CD4+ T cell response with different treatment by immunofluorescence.** Immunofluorescence staining of CD3^+^ CD4^+^ T cells in tumor sections after different treatments (n=5). Data are expressed as mean ± SEM, one-way ANOVA, **p*＜0.05, Exo^Ce6^+US, Exo^R848^, Exo^Ce6+R848^+US versus Exo^Ctrl^ ; ^#^*p*＜0.05, Exo^Ce6+R848^+US versus Exo^R848^.

**Supplemental Tables**

Table S1 Primers used in the study.

| Gene | Forward primer (5’-3’) | Reverse primer (5’-3’) |
| --- | --- | --- |
| *Ifn-γ* | ATGAACGCTACACACTGCATC | CCATCCTTTTGCCAGTTCCTC |
| *Tnf-α* | CCGGGAGAAGAGGGATAGCTT | TCGGACAGTCACTCACCAAGT |
| *Il-6* | TAGTCCTTCCTACCCCAATTTCC | TTGGTCCTTAGCCACTCCTTC |
| *Il-10* | GCTCTTACTGACTGGCATGAG | CGCAGCTCTAGGAGCATGTG |
| *Tgf-β* | CTCCCGTGGCTTCTAGTGC | GCCTTAGTTTGGACAGGATCTG |
| *Gapdh* | TGGATTTGGACGCATTGGTC | TGGATTTGGACGCATTGGTC |
| *Il-1β* | TGAAAACACAGAAGTAACGTCCG | CCCAGGAGGAAATTGTAATGGGA |
| *Il-12* | TGGTTTGCCATCGTTTTGCTG | ACAGGTGAGGTTCACTGTTTCT |
| *iNos* | GTTCTCAGCCCAACAATACAAGA | GTGGACGGGTCGATGTCAC |
| *Arg-1* | CTGAGAGATTCAAGGCAAGAGG | GAACGCGCTATCTTACCCCAG |
| *Mrc-1* | AAACACAGACTGACCCTTCCC | GTTAGTGTACCGCACCCTCC |
| *Fizz-1* | CCAATCCAGCTAACTATCCCTCC | ACCCAGTAGCAGTCATCCCA |
| *cel-miR-54-5p*  *U6* | AGGATATGAGACGACGAGAACA  CTCGCTTCGGCAGCACA | Provided in the kit  Provided in the kit |
